# Mitogenomic Insights into Temperature Adaptation: A Comparative Study of the Subfamily Corydalinae Davis, 1903 (Megaloptera: Corydalidae)

**DOI:** 10.3390/insects16111151

**Published:** 2025-11-10

**Authors:** Wenye Wang, Shuo Tian, Zifan Wang, Yuyu Wang, Xingyue Liu, Yunlan Jiang

**Affiliations:** 1 College of Plant Protection, Hebei Agricultural University, Baoding 071001, China; wenye_w1004@163.com (W.W.); shuotian139@126.com (S.T.); zifan_wang2025@163.com (Z.W.); wangyy_amy@126.com (Y.W.); 2 Institute of Zoology, Chinese Academy of Sciences, Beijing 100101, China; 3 Department of Entomology, China Agricultural University, Beijing 100193, China

**Keywords:** Corydalinae, mitochondrial genomes, comparative analyses, temperature adaptation

## Abstract

Corydalinae (dobsonfly) is one of the subfamilies of the megalopteran family Corydalidae. Species of Corydalinae are some of the largest freshwater insects in the world. Comparative analyses of mitochondrial genomes in Corydalinae were conducted. The evolutionary rates of 13 PCGs of fifty species in Corydalinae were estimated. The total PCGs of Corydalinae exhibited negative AT bias, ranging from −0.1810 to −0.1408. The Ka/Ks ratio of total PCGs in Corydalinae ranged from 0.1011 (*Protohermes*) to 0.1673 (*Chloroniella*). Phylogenetic analysis of Corydalinae was conducted using mitogenomes. Positive selection analyses, conducted based on the Corydalinae phylogenetic topology, revealed potential positively selected sites in the genera *Acanthacorydalis*, *Corydalus*, *Neoneuromus*, *Nevromus*, and *Protohermes*. These genera were distributed in areas characterized by marked seasonality and pronounced annual thermal amplitude variations. The observed divergence in potential positive selection sites and evolutionary rates might be attributed to differential adaptive evolution in response to climatic factors, potentially reflecting distinct molecular mechanisms underlying species-specific adaptation to rapid environmental shifts.

## Simple Summary

Corydalinae Davis, 1903, is a subfamily of Corydalidae in the order Megaloptera with 177 dobsonfly species. Larvae of Corydalinae are predatory and inhabit lotic habitats in montane areas. Mitochondrial genes might be exposed to differential selective pressures across populations or species exhibiting divergent biogeographical distributions or occupying distinct ecological niches. Comparative analyses of mitochondrial genomes were conducted using all published mitochondrial genomes of Corydalinae in this study. Non-synonymous to synonymous substitution rate ratio (Ka/Ks) analysis suggested that total protein-coding genes (PCGs) across species from eight genera of Corydalinae might be under purifying selection. Positive selection pressure analysis revealed potential positive selection sites in mitochondrial genes of five genera distributed in areas with significant annual temperature difference, which may be attributed to differential adaptive evolution in response to climatic factors.

## 1. Introduction

Mitochondrial DNA has been used extensively in studies on molecular phylogeny, evolutionary history, biogeographical history, and population phylogeographic pattern due to its strict maternal inheritance and highly conserved features [1,2,3,4,5,6,7]. Mitochondrial genes, as essential components of enzymes responsible for energy and heat production, have a major influence on enabling organisms to adapt to environmental temperature changes [8,9]. Mitochondria exhibit a capacity to adapt to stress through dynamic regulatory mechanisms that preserve cellular energy homeostasis. Such adaptation involves coordinated molecular mechanisms operating at multiple hierarchical levels, including transcriptional regulation of stress-responsive genes, post-translational modulation of protein abundance/stability, and fine-tuning of enzymatic activity in metabolic pathways [10,11,12,13,14]. Mitochondrial genes might be exposed to differential selective pressures across populations or species exhibiting divergent biogeographical distributions or occupying distinct ecological niches [15]. The sequence variation at specific amino acid residues in mitochondrial genome-encoded cytochrome c oxidase (cox) subunits might enhance mitochondrial respiratory efficiency under hypoxic conditions, representing a putative molecular mechanism underlying high-altitude adaptation in animal species [16,17]. Previous studies suggested that mitochondrial gene expression could be influenced by environmental stress (e.g., climate, temperature). For instance, the expression of the *cox1* gene in *Dryophytes versicolor* was found to be sharply reduced under cold stress conditions [18]. Similarly, a study on expression patterns of mitochondrial genes in adapting to changes in temperature revealed that the expression of *cox3*, *nad2*, *nad4*, and *nad6* was markedly reduced under cold stress [19]. Analyses integrating environmental variables with evolutionary rates demonstrated significant temperature-related effects on *atp6*, *atp8*, *cox3*, and *nad1* [20]. It was reported that the mitochondrial genes *atp6*, *cytb*, *cox1*, and *nad4* in humans underwent climate-driven adaptive evolution [21,22]. Furthermore, it was found that *cox1*, *nad5* and *nad6* in Australian passerine were subject to selective pressure from climatic conditions [23].

The subfamily Corydalinae belongs to the megalopteran family Corydalidae and currently includes 177 species classified into nine genera, occurring in the Americas, parts of Asia, and southern Africa [24]. Larvae are entirely aquatic, with most species inhabiting lotic systems such as streams and rivers, whereas some occupy lentic bodies of water, generally lakes [25,26]. A consistent topology was obtained in phylogenetic analyses based on both morphological characteristics and mitogenomes, indicating that this subfamily can be divided into three major clades: the *Protohermes* clade (*Protohermes* + *Neurhermes*), the *Nevromus* clade (*Nevromus* + *Neoneuromus*), and the *Corydalus* clade (*Acanthacorydalis* + (*Platyneuromus*+ (*Chloronia* + *Corydalus*))). The *Nevromus* clade and the *Corydalus* clade were sister groups, and *Chloroniella* was recovered as the sister group to the remaining genera [6,27,28,29,30,31,32,33].

Mitochondrial genomes of forty-nine Corydalinae species have been published. Comparative analyses of these mitogenomes within the subfamily of Corydalinae were conducted in this study. The phylogeny of Corydalinae was reconstructed using Maximum Likelihood (ML) and Bayesian Inference (BI) based on four datasets. Positive selection analyses were conducted in the context of the Corydalinae phylogenetic topology.

## 2. Materials and Methods

### 2.1. Sequencing and Assembly

The voucher specimen of *Acanthacorydalis yunnanensis* was collected by Fan Yang on 15 June 2015 and preserved in 95% ethanol at Hebei Agricultural University (HEBAU), Baoding, China. Total genomic DNA was extracted from the thoracic muscle tissue using a TIANamp Genomic DNA Kit (TIANGEN Inc., Beijing, China). The mitochondrial genome was sequenced on an Illumina NovaSeq platform. Adapter sequences were removed from raw reads with Trimmomatic [34], and clean reads were assembled in GENEIOUS v9.0 [35] by mapping to the mitochondrial genome of *Acanthacorydalis orientalis* (GenBank accession: KF840564). Assembly parameters were set to a minimum overlap identity of 95%, a maximum of four ambiguities, and a minimum overlap length of 25 bp, with other settings kept at default. The other genomes of Corydalinae used in comparative analyses and phylogeny were downloaded from the National Center of Biotechnology Information (NCBI; https://www.ncbi.nlm.nih.gov/, accessed on 23 October 2024).

### 2.2. Bioinformatic Analysis of Mitogenomes

The tRNAs of *A. yunnanensis* were predicted by the tRNAscan-SE Search server (http://lowelab.ucsc.edu/tRNAscan-SE/index.html, accessed on 17 December 2023) [36]. The PCGs and rRNAs of *A. yunnanensis* were annotated by aligning them with other *Acanthacorydalis* species [6,32]. Nucleotide composition was determined using MEGA 7.0 [37]. AT-skew and GC-skew were calculated by AT-skew = (A − T)/(A + T) and GC-skew = (G − C)/(G + C), and the results were clustered and plotted in a figure with R programming language 4.3.3 [38].

In neutrality plot analysis, the difference in G + C content between the third codon position (GC3) and the average of the first and second positions (GC12) was examined to determine the relative effects of mutation pressure and natural selection on codon usage bias [39]. GC12 and GC3 values were calculated by MEGA 7.0 [37]. The neutrality plot was plotted with GC3 as the X-axis and GC12 as the Y-axis, and a linear regression equation was constructed. In the plot, a regression slope (regression coefficient) approaching 0 indicated that natural selection was the dominant force, whereas a slope approaching 1 indicated complete neutrality, with mutation pressure predominating [40]. The correlation coefficient was calculated using the correlation function, with the formula of $Correl\;\left(X,Y\right)=\frac{\sum_{i=1}^{n}\left(X_{i}-\bar{X}\right)\left(Y_{i}-\bar{Y}\right)}{n-1}$. When GC12 and GC3 showed a statistically significant correlation and the coefficient was close to 1, mutational neutrality predominated; in contrast, coefficients near 0 reflected a dominant effect of selection.

In DnaSP v6.12.03 [41], pairwise Ka/Ks values between every two species were obtained, with “first site” designated as the protein-coding region and nucleotide format defined as “mtDNA Drosophila Mitochondrial”. The average Ka/Ks values between each species and the other species were calculated as the final value for comparative analysis. The sequence saturation of PCGs was calculated by DAMBE v. 7.3.32 [42].

Tandem repeats in the complete control regions of 43 species were predicted using the web tool Tandem Repeats Finder v. 4.09 (https://tandem.bu.edu/trf/trf.html, accessed on 5 April 2025) and MISA-webtool v. 2.1 (https://webblast.ipk-gatersleben.de/misa/, accessed on 5 April 2025) [43,44].

### 2.3. Phylogenetic Analysis

For the phylogenetic analyses, fifty species of Corydalinae were included as ingroups, and five species (*Dysmicohermes ingens*, *Neochauliodes meridionalis*, *Protochauliodes humeralis*, *Parachauliodes buchi* and *Sialis jiyuni*) were used as outgroups (Table S1).

Amino acid sequences of the PCGs were aligned in MEGA 7.0 [37], and nucleotide sequences of rRNAs were aligned with an automatic algorithm in MAFFT 7.313 [45]. Phylogenetic trees were constructed based on four datasets: (i) matrix PCG123 containing nucleotide sequences of 13 PCGs; (ii) matrix PCG123 + rRNA containing nucleotide sequences of 13 PCGs and 2 rRNAs; (iii) matrix PCG12 + rRNA containing nucleotide sequences of 13 PCGs without the third codon position and 2 rRNAs; (iv) matrix PCGAA containing amino acid sequences of the 13 PCGs. PartitionFinder2 was used to determine the most suitable partitioning strategy and corresponding evolutionary model for each partition using the corrected Akaike Information Criterion (AICc) [46]. The final partitioning strategies for all datasets are presented in Table S2. BI analyses were conducted using MrBayes 3.2.7, following the partition model determined by PartitionFinder2 [47]. Two independent runs comprising four chains each were performed concurrently for two million generations (printfreq = 1000; burninfrac = 0.25). Analyses were considered converged when the standard deviation of split frequencies fell below 0.01. ML analyses were conducted using the IQ-TREE web server (http://iqtree.cibiv.univie.ac.at, accessed on 28 October 2024) with 1000 bootstrap replicates [48].

### 2.4. Detecting Selective Pressure

EasyCodeML, an interactive visual platform based on CodeML, was used to assess selective pressures in mitochondrial genomes [49,50]. A multiple sequence alignment of 13 PCGs from 50 species of Corydalinae was generated, comprising 3701 amino acid sites. Analyses were conducted using the branch-site model, which posited that selective pressures vary among different sites and different lineages by accounting for differences in ω values. Model comparisons were conducted between Model A and Model A null. Additionally, the Bayesian empirical Bayes (BEB) method was applied to estimate the posterior probability for each amino acid site being under positive selection in every lineage. Sites showing BEB posterior probabilities > 0.95 were inferred as positively selected, whereas those with BEB posterior probabilities > 0.99 were assigned higher confidence due to stronger statistical support. Maximum and minimum temperatures across the distribution areas of Corydalinae over the past five years were obtained from the NASA POWER platform (https://power.larc.nasa.gov/data-access-viewer/, accessed on 31 October 2025) [31,51].

## 3. Results

### 3.1. Protein-Coding Genes

**Figure 1:**
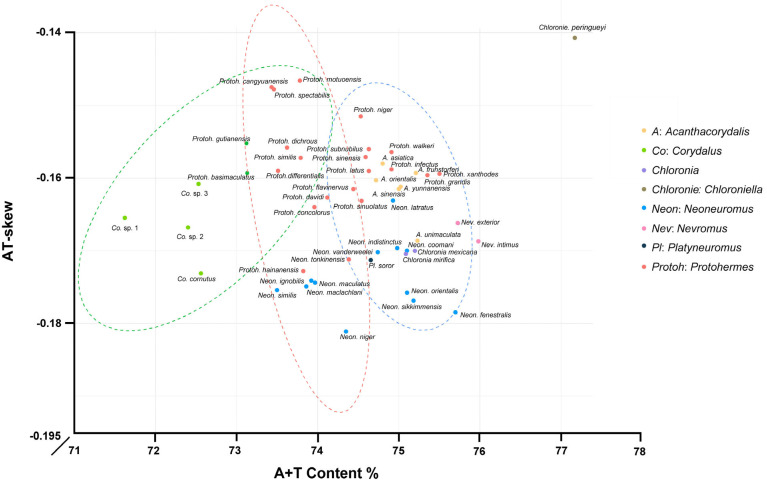
The A + T composition and AT-Skew in PCGs of Corydalinae (The dotted lines represent the clustering results).

**Figure 2:**
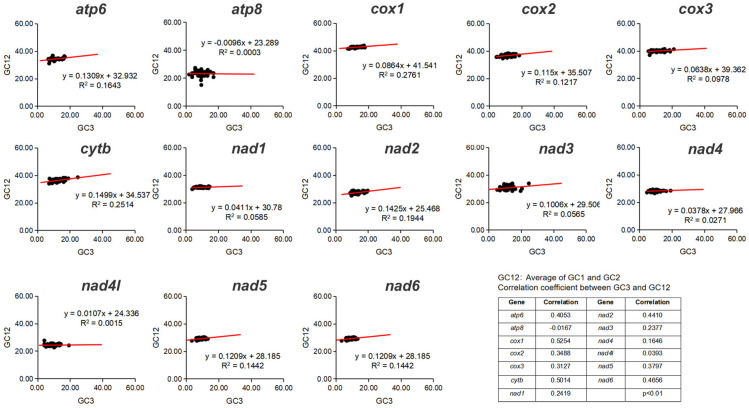
Neutrality plot of PCGs of the studied species of Corydalinae.

The protein-coding genes in Corydalinae were characterized by a negative AT-skew (−0.1613) and a marginally positive GC-skew (0.0003) (Figure 1, Table S3). Across the 13 PCGs, A + T content ranged from 71.70% (*Corydalus* sp. 1) to 77.19% (*Chloroniella peringueyi*). The neutrality plot analysis of 50 species of Corydalinae was conducted in this study. The correlation value showed a relatively significant positive correlation between GC12 and GC3 in *cox1* and *cytb* (correlation > 0.5), indicating that the entire codon position in these two genes might be influenced by mutation force (GC mutation bias). Furthermore, the regression coefficients of 13 PCGs were below 0.5, highlighting the dominant role of natural selection in shaping codon usage bias (Figure 2).

**Figure 3:**
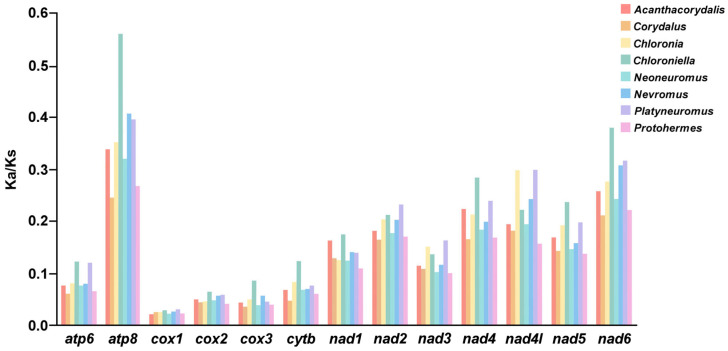
Ka/Ks values of the 13 PCGs of eight genera in Corydalinae.

The Ka/Ks ratio of the total PCGs and each PCG of 50 species in Corydalinae was analyzed (Figure 3 and Figure S1). The Ka/Ks values of the total PCGs of Corydalinae ranged from 0.0904 (*Protohermes gutianensis*) to 0.1662 (*Chloroniella peringueyi*), indicating that all the PCGs of Corydalinae might be under purifying selection (Figure S1). The Ka/Ks values of each PCG in eight genera were lower than 1, and those of *cox1* were the lowest among all 13 PCGs, meaning that the evolution rate of *cox1* was the slowest (Figure 3).

In the sequence saturation test, the index of substitution saturation (*Iss*) of the total PGCs and each site of the PCGs was significantly lower than the index of substitution saturation—critical *Iss* value (*Iss.c*), which suggested that the substitutions at all codon positions in the mitochondrial PCGS of *Acanthacorydalis* were not saturated (Table 1).

### 3.2. Non-Coding Regions

**Figure 4:**
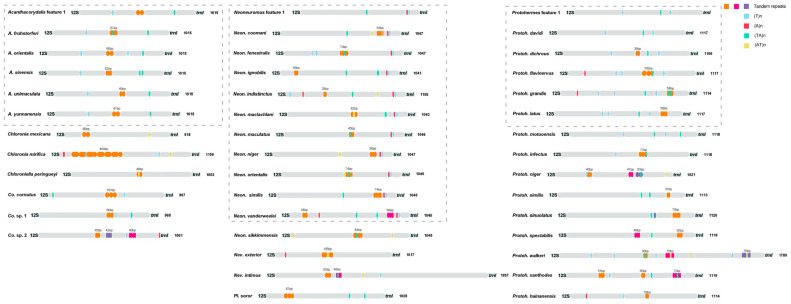
Structural features of mitochondrial control regions in Corydalinae. The locations and copy numbers of tandem repeats are represented in purple, pink, and orange. Blue boxes represent T tandem repeat units. Red boxes represent A tandem repeat units. Green boxes represent TA tandem repeat units. Yellow boxes represent AT tandem repeat units. Species in the same dashed box possess imaginative features.

In mitochondrial genomes of Corydalinae, 43 complete control regions were examined, with the longest reaching 1857 bp (Figure 4). A + T content varied from 76.99% in *Nevromus intimus* to 91.18% in *Chloronia mexicana*. Analyses of 43 species revealed T, A, TA, and AT repeat units, tandem repeat fragments ranging from one to four repeats. In *Acanthacorydalis*, there was one T repeat unit, one repeat fragment, and one TA repeat unit in the control regions, and the repetitive characteristics were almost identical. One or two T repeat units and one TA repeat unit were found in six species of *Protohermes* (*Protoh. davidi*, *Protoh. dichrous*, *Protoh. flavinervus*, *Protoh. grandis*, *Protoh. Latus*, and *Protoh. motuoensis*). All species in *Neoneuromus* contain at least one set of tandem repeat segments in the control regions. There were similar features in the control regions of ten *Neoneuromus* species (excluding *Neon. sikkimmensis*) concluding one set of TA repeat unit, one A repeat unit, and one T repeat unit.

### 3.3. Phylogenetic Analysis of Corydalinae

**Figure 5:**
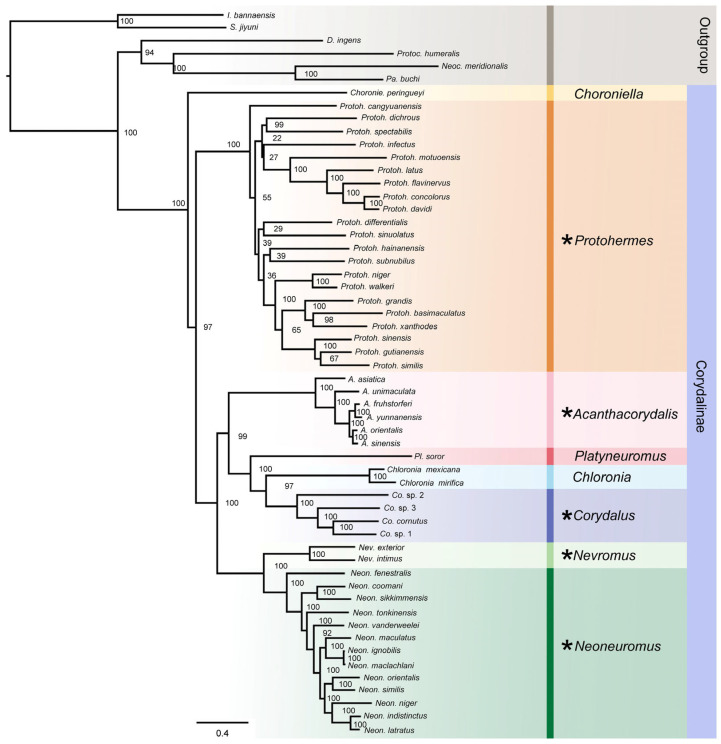
ML tree of the subfamily Corydalinae recovered using PCG123+2 rRNA matrix. The bootstrap values of the corresponding nodes are shown. Genera with positive selection sites in positive selection analysis are represented by asterisks.

Bayesian Inference and Maximum Likelihood analyses using four concatenated datasets yielded phylogenetic trees with consistent topology (Figure 5). The subfamily Corydalinae was divided into four clades. *Chloroniella* was located at the most basal part of Corydalinae, and *Protohermes* was recovered as the sister group to all remaining genera. *Acanthacorydalis* was recovered as the sister group to *Platyneuromus* + (*Chloronia* + *Corydalus*). The *Nevromus* branch (*Nevromus* + *Neoneuromus*) was the sister group to the *Corydalus* branch. Notably, analyses of the PCGAA dataset (BI and ML) placed *Acanthacorydalis* as sister to *Nevromus* + *Neoneuromus*. ML analysis of the PCGAA dataset and BI analyses of the PCGAA and PCG123 datasets consistently recovered *Protohermes* as a sister group to *Chloroniella*.

### 3.4. Results of Detecting Selective Pressure

Based on the phylogeny inferred from the ML analyses, a branch-site model was used for positive selection analysis of eight genera in Corydalinae. The likelihood ratio test (LRT) was used to compare Model A with the corresponding Model A null, yielding *p* < 0.05. It showed there were in total 16 potential positive selection sites (BEB posterior probabilities > 0.95) in five genera: *Acanthacorydalis* (1 site), *Corydalus* (8 sites), *Neoneuromus* (1 site), *Nevromus* (2 sites), and *Protohermes* (4 sites). And the sites were distributed in different genes: *Acanthacorydalis* (*nad2*), *Corydalus* (*atp8*, *cox1*, *nad2*, *nad4*, and *nad6*), *Neoneuromus* (*nad1*), *Nevromus* (*atp8* and *nad4L*), and *Protohermes* (*nad2*, *nad4*, and *nad5*) (Table 2). Temperature ranges across the distribution areas of Corydalinae revealed that the five genera (*Acanthacorydalis*, *Corydalus*, *Neoneuromus*, *Nevromus*, *Protohermes*) exhibited broader annual temperature variations and could adapt to minimum temperatures of −20 °C (Table 3 and Table S4).

## 4. Discussion

Comparative analyses of mitochondrial genomes of Corydalinae were constructed in this study. The mitogenomes of Corydalinae showed negative AT-skew, which was consistent with previous studies in Megaloptera, such as *Acanthacorydalis orientalis*, *Protohermes concolorus*, *Neoneuromus bowringi*, *Nevromus exterior*, and *Protohermes biconicus* [3,52,53,54,55,56]. Some mitogenomes of Lepidoptera, Diptera, Hemiptera, and Venerida [57,58,59,60] also showed negative AT-skew, while that of the genus *Neoneuromus* showed positive AT-skew [20].

The Ka/Ks ratio of Corydalinae was less than 1, indicating that the total PCGs in eight genera might be subject to purifying selection, which was consistent with previous studies [20]. Analyses of the Ka/Ks ratio were widely used to detect changes in selection intensity. However, this approach cannot detect variation in selection acting on individual sites within protein-coding sequences [61]. The branch-site models enabled the detection of positively selected sites in phylogenetic branches by permitting the ω ratios to vary across both branches and sites [62].

Selection pressure analyses were conducted on eight genera of Corydalinae. It showed that there were positive selection signals in five genera (*Acanthacorydalis*, *Corydalus*, *Neoneuromus*, *Nevromus*, and *Protohermes*), and evolutionary rates (Ka/Ks < 1) revealed that the majority of genes were predominantly subject to purifying selection (Figure 3 and Figure 5). Genera *Acanthacorydalis*, *Protohermes*, and *Neoneuromus* are all distributed in the Palearctic and Oriental realm, including temperate and subtropical climates. *Nevromus* is distributed in Nepal, where the climate exhibits high climatic diversity. *Corydalus* is distributed in the Nearctic/Neotropical transition zone, mainly on the American continent, especially in the eastern and central regions of North America, diverse climates which include temperate, subtropical, and tropical climates. Climate type appears to be a key factor for species with narrow geographic ranges; for example, *Co. magnus* prefers cold temperate climates, while *Co. mirifica* favors tropical climates [63]. Although *Chloronia* was also distributed in the Nearctic and Nearctic/Neotropical regions, its occurrence is restricted to the semi-warm and warm climates of southeastern Mexico in the New North region [63]. The five genera exhibiting signals of positive selection were distributed in areas characterized by pronounced seasonal climatic shifts and could adapt to extreme low temperatures (−20 °C).

Mitochondrial genes might be subject to differential evolutionary pressures among populations or species distributed in varied habitats. Mitochondrial genes exhibited signatures of positive selection associated with climatic variables [64]. Temperature seasonality, wet month precipitation, and precipitation seasonality were identified as key factors shaping adaptive evolution [63]. It was suggested that mitochondrial genes exhibited different evolutionary rates under various environmental conditions in previous studies. In this study, the potential sites subject to positive selection appear to be associated with differential adaptive evolution in response to climatic factors. potentially reflecting distinct molecular mechanisms underlying species-specific adaptation to rapid environmental shifts.

## 5. Conclusions

Comparative mitochondrial genome analysis of Corydalinae revealed that the evolution of PCGs was under purifying selection, with Ka/Ks < 1 for most species. Endemic to South Africa, the genus *Chloroniella* displays elevated Ka/Ks ratios for *atp8*, *nad4*, *nad5*, and *nad6* relative to other genera. This may reflect evolutionary divergence among species or stem from its confinement to South Africa, where distinctive environmental and climatic conditions prevail, unlike the diverse climates inhabited by other genera. Positive selection analysis in Corydalidae revealed putative selection sites in five genera inhabiting regions north of the Tropic of Cancer, characterized by pronounced seasonal temperature fluctuations. The difference in potential positive selection sites and evolutionary rates may be attributed to differential adaptive evolution in response to climate change, potentially reflecting distinct molecular mechanisms underlying species-specific adaptation to rapid environmental shifts.

## Figures and Tables

**Table 1:** Sequence saturation test results for different codon positions of the PCGs in the dataset. P = probability, *Iss.cSym/Iss.cAsym* = *Iss.c* for symmetrical/asymmetrical topology.

Codon Position	*Iss*	*Iss.cSym*	*P cSym*	*Iss.cAsym*	*P cAsym*
1st position	0.530	0.849	0.000	0.837	0.000
2nd position	0.142	0.842	0.000	0.758	0.000
3rd position	0.057	0.849	0.000	0.837	0.000
All positions	0.221	0.858	0.000	0.846	0.000

**Table 2:** Branch-site model parameters and results for Corydalinae (Model A vs. null Model A; *p*-value < 0.05 indicates statistical significance).

Genus	LRT *p*-Value	Positive Sites	Genes
*Acanthacorydalis*	0.000000023	2190 E 0.972 *	*nad2*
*Corydalus*	0.000010332	260 S 0.981 *, 751 T 0.964 *, 1913 S 0.991 **, 2040 E 0.968 *, 2300 M 0.978 *, 2826 V 0.981 *, 3600 N 0.988 *, 3631 E 0.959 *	*atp8*, *cox1*, *nad2*, *nad4*, *nad6*
*Neoneuromus*	0.000000364	1835 A 0.950 *	*nad1*
*Nevromus*	0.000000017	267 N 0.969 *, 2906 N 0.957 *	*atp8*, *nad4l*
*Protohermes*	0.000000048	2056 D 0.960 *, 2754 C 0.987 *, 2782 S 0.987 *, 3356 S 0.984 *	*nad2*, *nad4*, *nad5*

Note: * and ** indicate BEB posterior probability values > 0.95 and >0.99.

**Table 3:** Maximum and minimum temperatures for the distribution areas of Corydalinae over the past five years (2020–2024).

Genus	Distribution	Highest Temperature (°C)	Lowest Temperature (°C)
*Acanthacorydalis*	Oriental Region, Northeastern India to Vietnam and China	36.51–40.19 (Northeast India)	−31.32–−26.18 (Northeast China)
*Chloronia*	Neotropical Region, Brazil through Mexico, Lesser Antilles	37.21–42.49 (Amazon Basin)	0.39–1.93 (Amazon Basin)
*Chloroniella*	South Africa	41.96–45.46	4.3–9.43
*Corydalus*	North, Central, and South America	37.21–42.49 (Amazon Basin)	−44.62–−39.48 (South-central Canada)
*Neoneuromus*	Oriental Region, Northeastern India to Malay Peninsula and China	36.51–40.19 (Northeast India)	−31.32–−26.18 (Northeast China)
*Nevromus*	Oriental Region, Northwest India to Indonesia and China	44.58–48.42 (Northwest India)	−31.32–−26.18 (Northeast China)
*Platyneuromus*	Central America to Northeastern Mexico	43.38–46.84 (Northeast Mexico)	2.71–6.01 (Northeast Mexico)

## Data Availability

The data supporting the findings of this study are openly available from the National Center for Biotechnology Information at https://www.ncbi.nlm.nih.gov (accessed on 15 June 2024), accession number PP968713.

## Supplementary Materials

The supporting data for this study can be obtained from https://www.mdpi.com/article/10.3390/insects16111151/s1: Figure S1: Ka/Ks values of the total PCGs of 48 species of Corydalinae; Figure S2: ML tree of the subfamily Corydalinae estimated using PCG123 matrix. The bootstrap values of the corresponding nodes are shown in the figure; Figure S3: BI tree of the subfamily Corydalinae estimated using PCG123 matrix. The support values of the corresponding nodes are shown in the figure; Figure S4: ML tree of the subfamily Corydalinae estimated using PCG12+2 rRNA matrix. The bootstrap values of the corresponding nodes are shown in the figure; Figure S5: BI tree of the subfamily Corydalinae estimated using PCG12+2 rRNA matrix. The support values of the corresponding nodes are shown in the figure; Figure S6: BI tree of the subfamily Corydalinae estimated using PCG123+2 rRNA matrix. The support values of the corresponding nodes are shown in the figure; Figure S7: ML tree of the subfamily Corydalinae estimated using PCGAA matrix. The bootstrap values of the corresponding nodes are shown in the figure; Figure S8: BI tree of the subfamily Corydalinae estimated using PCGAA matrix. The support values of the corresponding nodes are shown in the figure; Table S1: Taxonomic groups employed in the phylogenetic analyses; Table S2: Partitioning schemes and the best-fitting models for each subset; Table S3: Nucleotide composition and skews in the total PCGs of Corydalinae; Table S4. Details of temperature range of the distribution of Corydalinae during 2020–2024. References [5,20,33,51,65,66,67] are cited in the Supplementary Materials.
