# Supplementary material for: Mitogenomic Insights into Temperature Adaptation: A Comparative Study of the Subfamily Corydalinae Davis, 1903 (Megaloptera: Corydalidae)
Source: Insects. 2025 Nov 10;16(11):1151. doi: 10.3390/insects16111151 (PMC12653108; doi:10.3390/insects16111151)
Supplement: Supplementary file 1 [file insects-16-01151-s001.zip › Table Supplementary.pdf]

Table S1. Taxonomic groups employed in the phylogenetic analyses.

Family	Subfamily	Genus	Species	Accession number	Reference
Corydalidae	Corydalinae	*Acanthacorydalis*	*A. yunnanensis*	PP968713	This study
			A. asiatica	MW642319	Jiang *et al*., 2022
			A. fruhstorferi	MW642265	Wang *et al*., 2014
			A. orientalis	KF840564	Jiang *et al*., 2022
			A. sinensis	MW642266	Jiang *et al*., 2022
			A. unimaculata	MW642267	Jiang *et al*., 2022
		Chloronia	*Chloronia mexicana*	MW642268	Jiang *et al*., 2022
			Chloronia mirifica	KU302713	Jiang *et al*., 2015
		Chloroniella	*Chloronie. peringueyi*	MW642269	Jiang *et al*., 2022
		Corydalus	*Co. cornutus*	FJ171323	Beckenbach *et al*., 2022
			Co. sp. 1	MW642270	Jiang *et al*., 2022
			Co. sp. 2	MW642271	Jiang *et al*., 2022
			Co. sp. 3	MW642301	Jiang *et al*., 2022
		Neoneuromus	*Neon. coomani*	MW965203	Tu *et al*., 2021
			Neon. fenestralis	MW965199	Tu *et al*., 2021
			Neon. ignobilis	MW642302	Jiang *et al*., 2022
			Neon. indistinctus	MW965202	Tu *et al*., 2021
			Neon. latratus	MW965206	Tu *et al*., 2021
			Neon. maclachlani	MW965201	Tu *et al*., 2021
			Neon. maculatus	MW965197	Tu *et al*., 2021
			Neon. niger	MW965196	Tu *et al*., 2021
			Neon. orientalis	MW965205	Tu *et al*., 2021
			Neon. sikkimmensis	MW965204	Tu *et al*., 2021
			Neon. similis	MW965198	Tu *et al*., 2021
			Neon. tonkinensis	KP126231	Jiang *et al*., 2015
			Neon. vanderweelei	MW965200	Tu *et al*., 2021
		Nevromus	*Nev. exterior*	KP126232	Jiang *et al*., 2015
			Nev. intimus	NC072694	Hassan *et al*., 2022
		Platyneuromus	*Pl. soror*	MW642273	Jiang *et al*., 2022
		Protohermes	*Protoh. basimaculatus*	MW642274	Jiang *et al*., 2022
			Protoh. cangyuanensis	MW642275	Jiang *et al*., 2022
			Protoh. concolorus	EU526394	Hua *et al*., 2009
			Protoh. davidi	MW642277	Jiang *et al*., 2022
			Protoh. dichrous	MW642278	Jiang *et al*., 2022
			Protoh.differentialis	MW642303	Jiang *et al*., 2022
			Protoh. flavinervus	MW642279	Jiang *et al*., 2022
			Protoh. grandis	MW642280	Jiang *et al*., 2022
			Protoh. gutianensis	MW642304	Jiang *et al*., 2022
			Protoh. hainanensis	MW642281	Jiang *et al*., 2022
			Protoh. infectus	MW642282	Jiang *et al*., 2022

			Protoh. latus	MW642283	Jiang *et al*., 2022
			Protoh. motuoensis	MW642284	Jiang *et al*., 2022
			Protoh. niger	MW642285	Jiang *et al*., 2022
			Protoh. similis	MW642286	Jiang *et al*., 2022
			Protoh. sinensis	MW642305	Jiang *et al*., 2022
			Protoh. sinuolatus	MW642287	Jiang *et al*., 2022
			Protoh. spectabilis	MW642288	Jiang *et al*., 2022
			Protoh. subnubilus	MW642306	Jiang *et al*., 2022
			Protoh. xanthodes	MW642289	Jiang *et al*., 2022
			Protoh. walkeri	ON332530	Hassan *et al*., 2022
	Chauliodinae	*Dysmicohermes*	*D. ingens*	KJ806318	Wang *et al*., 2014
		Neochauliodes	*Neoc. meridionalis*	MW642293	Jiang *et al*., 2022
		Parachauliodes	*Pa. buchi*	MT232266	Jiang et al., 2022
		Protochauliodes	*Protoc. humeralis*	MW642313	Jiang *et al*., 2022
Sialidae		*Indosialis*	*I. bannaensis*	MW642316	Jiang *et al*., 2022
		Sialis	*S. jiyuni*	MW642261	Jiang *et al., 2022*

Table S2. Partitioning schemes and the best-fitting models for each subset.

matrix	Subset	Best model	Subset sites	Alignment
PCG123	1	GTR+I+G	1-669\3 3817-4947\3;	atp6_pos1, cytb_pos1
	2	GTR+I+G	2-669\3 3032-3816\3 827-2355\3;	atp6_pos2, cox3_pos2, cox1_pos2
	3	GTR+I+G	3-669\3 2358-3030\3 6915-7260\3 10596-11103\3;	atp6_pos3, cox2_pos3, nad3_pos3, nad6_pos3
	4	GTR+I+G	670-825\3 10594-11103\3 5896-6912\3 6913-7260\3;	atp8_pos1, nad6_pos1, nad2_pos1, nad3_pos1
	5	GTR+I+G	671-825\3 10595-11103\3 3818-4947\3 2357-3030\3 5897-6912\3 6914-7260\3;	atp8_pos2, nad6_pos2, cytb_pos2, cox2_pos2, nad2_pos2, nad3_pos2
	6	GTR+G	5898-6912\3 828-2355\3 672-825\3;	nad2_pos3, cox1_pos3, atp8_pos3
	7	GTR+I+G	826-2355\3 3031-3816\3 2356-3030\3;	cox1_pos1, cox3_pos1, cox2_pos1
	8	GTR+I+G	3819-4947\3 3033-3816\3;	cytb_pos3, cox3_pos3
	9	GTR+I+G	8884-10593\3 4948-5895\3 8599-8883\3 7261-8598\3;	nad5_pos1, nad1_pos1, nad4l_pos1, nad4_pos1
	10	GTR+I+G	7262-8598\3 8600-8883\3 4949-5895\3 8885-10593\3;	nad4_pos2, nad4l_pos2, nad1_pos2, nad5_pos2
	11	GTR+G	7263-8598\3 4950-5895\3 8886-10593\3 8601-8883\3;	nad4_pos3, nad1_pos3, nad5_pos3, nad4l_pos3
PCG123+rRNAs	1	GTR+I+G	1-771 772-2084;	12s, 16s
	2	GTR+I+G	7980-8996\3 2754-2909\3 12678-13187\3 8997-9344\3 2085-2753\3;	nad2_pos1, atp8_pos1, nad6_pos1, nad3_pos1, atp6_pos1
	3	GTR+I+G	4441-5114\3 5902-7031\3 2086-2753\3 5116-5900\3 2911-4439\3;	cox2_pos2, cytb_pos2, atp6_pos2, cox3_pos2, cox1_pos2
	4	GTR+I+G	2087-2753\3 5903-7031\3 12680-13187\3 8999-9344\3;	atp6_pos3, cytb_pos3, nad6_pos3, nad3_pos3
	5	GTR+I+G	7981-8996\3 2755-2909\3 12679-13187\3;	nad3_pos2, nad2_pos2, atp8_pos2, nad6_pos2
	6	GTR+I+G	5117-5900\3 2912-4439\3 2756-2909\3;	cox2_pos3, cox3_pos3, cox1_pos3, atp8_pos3
	7	GTR+I+G	5115-5900\3 4440-5114\3 2910-4439\3 5901-7031\3;	cox3_pos1, cox2_pos1, cox1_pos1, cytb_pos1
	8	GTR+I+G	10968-12677\3 10683-10967\3 9345-10682\3 7032-7979\3;	nad5_pos1, nad4l_pos1, nad4_pos1, nad1_pos1
	9	GTR+I+G	9346-10682\3 10684-10967\3 10969-12677\3 7033-7979\3;	nad4_pos2, nad4l_pos2, nad5_pos2, nad1_pos2
	10	GTR+G	7034-7979\3 9347-10682\3	nad1_pos3, nad4_pos3,

			10685-10967\3 10970-12677\3;	nad4l_pos3, nad5_pos3
	11	GTR+G	7982-8996\3;	nad2_pos3
PCG12+rRNAs	1	GTR+I+G	1-771 772-2084;	12s, 16s
	2	GTR+I+G	4629-5382\2 2085-2530\2;	cytb_pos1, atp6_pos1
	3	GTR+I+G	4630-5382\2 3656-4104\2 2086-2530\2 2636-3654\2 4106-4628\2;	cytb_pos2, cox2_pos2, atp6_pos2, cox1_pos1, cox3_pos2
	4	GTR+I+G	6693-6924\2 2531-2635\2 9147-9486\2 6015-6692\2;	nad3_pos1, atp8_pos1, nad6_pos1, nad2_pos1
	5	GTR+I+G	6016-6692\2 6694-6924\2 2532-2635\2 9148-9486\2;	nad2_pos2, nad3_pos2, atp8_pos2, nad6_pos2
	6	GTR+I+G	2637-3654\2 3655-4104\2 4105-4628\2;	cox1_pos2, cox2_pos1, cox3_pos1
	7	GTR+I+G	8007-9146\2 5383-6014\2 7817-8006\2 6925-7816\2;	nad5_pos1, nad1_pos1, nad4l_pos1, nad4_pos1
	8	GTR+I+G	7818-8006\2 6926-7816\2 5384-6014\2 8008-9146\2;	nad4l_pos2, nad4_pos2, nad1_pos2, nad5_pos2
PCG AA	1	LG+I+G	786-1010 1-223 1011-1272 276-785;	*cox2, atp6, cox3, cox1*
	2	LG+I+G	1966-2304 2421-2866 2867-2961 2305-2420 2962-3531 224-275 3532-3701;	*nad2, nad4, nad4l, nad3, nad5, atp8, nad6*
	3	LG+I+G	1650-1965 1273-1649;	*nad1, cytb*

Table S3. Nucleotide composition and skews in the complete PCGs of Corydalinae.

Species	T(U)	C	A	G	Total	A+T%	G+C%	AT-Skew	GC-Skew
A. fruhstorferi	43.6	12.3	31.6	12.4	11118.0	75.23	24.77	-0.1595	0.0044
A. orientalis	43.4	12.5	31.4	12.8	11118.0	74.74	25.26	-0.1605	0.0121
A. sinensis	43.6	12.4	31.4	12.6	11118.0	75.02	24.98	-0.1617	0.0090
A. unimaculata	44.0	12.1	31.3	12.6	11118.0	75.25	24.75	-0.1688	0.0196
A. yunnanensis	43.6	12.4	31.5	12.5	11118.0	75.05	24.95	-0.1613	0.0022
Chloronia mexicana	44.0	12.1	31.2	12.6	11121.0	75.25	24.75	-0.1699	0.0207
Chloronia mirifica	44.0	12.2	31.2	12.7	11121.0	75.12	24.88	-0.1702	0.0213
Chloronie. peringueyi	44.0	11.2	33.2	11.6	11115.0	77.19	22.81	-0.1408	0.0178
Co. cornutus	42.6	13.6	30.0	13.7	11121.0	72.64	27.36	-0.1728	0.0030
Co. sp. 1	41.8	14.4	29.9	13.9	11121.0	71.70	28.30	-0.1653	-0.0187
Co. sp. 2	42.3	13.9	30.2	13.7	11121.0	72.46	27.54	-0.1663	-0.0069
Co. sp. 3	42.1	13.7	30.5	13.7	11121.0	72.59	27.41	-0.1607	0.0007
Neon. coomani	44.0	12.3	31.1	12.6	11118.0	75.11	24.89	-0.1706	0.0148
Neon. fenestralis	44.6	11.9	31.1	12.3	11121.0	75.73	24.27	-0.1788	0.0174
Neon. ignobilis	43.4	12.7	30.5	13.3	11121.0	73.97	26.03	-0.1741	0.0211
Neon. indistinctus	43.9	12.5	31.1	12.5	11121.0	75.03	24.97	-0.1697	0.0004
Neon. latratus	43.6	12.4	31.4	12.6	11121.0	74.98	25.02	-0.1632	0.0050
Neon. maclachlani	43.4	12.8	30.5	13.3	11121.0	73.91	26.09	-0.1748	0.0172
Neon. maculatus	43.5	12.8	30.6	13.2	11121.0	74.01	25.99	-0.1743	0.0138
Neon. niger	43.9	12.5	30.5	13.1	11121.0	74.40	25.60	-0.1810	0.0228
Neon. orientalis	44.2	12.2	31.0	12.6	11121.0	75.15	24.85	-0.1760	0.0174
Neon. sikkimmensis	44.3	12.1	30.9	12.7	11121.0	75.22	24.78	-0.1775	0.0232
Neon. similis	43.2	13.1	30.3	13.4	11121.0	73.55	26.45	-0.1752	0.0116
Neon. tonkinensis	43.6	12.8	30.8	12.8	11121.0	74.43	25.57	-0.1714	0.0028
Neon. vanderweelei	43.8	12.6	31.0	12.7	11121.0	74.79	25.21	-0.1706	0.0036
Nev. exterior	44.2	11.7	31.6	12.6	11121.0	75.77	24.23	-0.1662	0.0375
Nev. intimus	44.4	11.5	31.6	12.5	11121.0	76.04	23.96	-0.1686	0.0439
Pl. soror	43.7	12.6	31.0	12.7	11121.0	74.70	25.30	-0.1701	0.0021
Protoh. basimaculatus	42.4	13.3	30.8	13.5	11124.0	73.20	26.80	-0.1598	0.0077
Protoh. cangyuanensis	42.2	13.4	31.3	13.1	11121.0	73.50	26.50	-0.1478	-0.0126
Protoh. concolorus	43.1	12.9	30.9	13.1	11124.0	74.03	25.97	-0.1645	0.0059
Protoh. davidi	43.1	12.7	31.0	13.1	11124.0	74.19	25.81	-0.1632	0.0136
Protoh. dichrous	42.6	13.2	31.1	13.2	11124.0	73.70	26.30	-0.1561	0.0000
Protoh. differentialis	42.7	13.3	30.9	13.1	11124.0	73.59	26.41	-0.1595	-0.0068
Protoh. flavinervus	43.3	12.5	31.2	12.9	11124.0	74.51	25.49	-0.1619	0.0155
Protoh. grandis	43.7	11.9	31.7	12.7	11124.0	75.41	24.59	-0.1601	0.0296
Protoh. gutianensis	42.3	13.5	30.9	13.3	11124.0	73.18	26.82	-0.1556	-0.0064
Protoh. hainanensis	43.3	12.9	30.6	13.2	11124.0	73.89	26.11	-0.1726	0.0141
Protoh. infectus	43.5	12.4	31.5	12.7	11124.0	74.98	25.02	-0.1596	0.0126
Protoh. latus	43.3	12.5	31.4	12.8	11124.0	74.69	25.31	-0.1595	0.0146
Protoh. motuoensis	42.4	12.9	31.5	13.3	11124.0	73.85	26.15	-0.1474	0.0134
Protoh. niger	43.0	12.6	31.6	12.8	11124.0	74.60	25.40	-0.1523	0.0092

Species	T(U)	C	A	G	Total	A+T%	G+C%	AT-Skew	GC-Skew
Protoh. similis	42.8	13.1	31.1	13.1	11124.0	73.85	26.15	-0.1579	0.0017
Protoh. sinensis	43.2	12.6	31.4	12.8	11124.0	74.65	25.35	-0.1582	0.0078
Protoh. sinuolatus	43.4	12.6	31.2	12.8	11124.0	74.60	25.40	-0.1640	0.0110
Protoh. spectabilis	42.2	13.4	31.3	13.1	11124.0	73.53	26.47	-0.1484	-0.0136
Protoh. subnubilus	43.2	12.6	31.5	12.7	11124.0	74.69	25.31	-0.1567	0.0071
Protoh. walkeri	43.4	12.3	31.6	12.7	11124.0	74.97	25.03	-0.1571	0.0180
Protoh. xanthodes	43.8	11.9	31.8	12.6	11124.0	75.57	24.43	-0.1596	0.0294

Table S4. Details temperature range of the distribution of Corydalinae during 2020–2024.

Genus	Distribution	Highest temperature	Lowest temperature
Acanthacorydalis	Vietnam	31.45 - 34.07	18.36 - 19.63
	Northeast China	31.11 - 34.56	–31.32 - –26.18
	Southern China	35.18 - 39.34	–8.12 - –4.09
	Laos	33.17 - 38.15	2.82 - 8.0
	Northeastern India	36.51 - 40.19	10.38 - 8.91
Chloronia			
	Amazon Basin	37.21 - 42.49	1.93 - 0.39
	Brazil	37.91 - 40.06	13.62 - 14.56
	Mexico	39.31 - 41.83	12.15 - 13.54
	Lesser Antilles	30.7 - 31.11	24.45 - 25.33
Chloroniella			
	South Africa (Cape Town)	21.49 - 23.75	10.08 - 13.18
	South Africa (Lowveld, Kruger NP)	41.96 - 45.46	4.3 - 9.43
Corydalus			
	south-central Canada	29.75 - 34.01	–44.62 - –39.48
	Guatemala	32.21 - 37.52	7.61 - 9.71
	Amazon Basin	37.21 - 42.49	1.93 - 0.39
Neoneuromus			
	Northeast India	36.51 - 40.19	10.38 - 8.91
	Malay Peninsula	30.55 - 32.38	17.1 - 20
	Northeast China	31.11 - 34.56	–31.32 - –26.18
Nevromus			
	northwest India	44.58 - 48.42	–0.28 - 3.32
	Indonesia	30.83 - 31.43	20.57 - 20.9
	Northeast China	31.11 - 34.56	–31.32 - –26.18
Platyneuromus			
	Central America	31.87 - 35.99	22.2 - 23.33
	northeast Mexico	43.38 - 46.84	2.71 - 6.01
Protohermes			
	northwest India	44.58 - 48.42	–0.28 - 3.32
	Indonesia	30.83 - 31.43	20.57 - 20.9
	Northeast China	31.11 - 34.56	–31.32 - –26.18

References

Beckenbach, A.T.; Stewart, B. Insect mitochondrial genomics 3: the complete mitochondrial genome sequences of representatives from two neuropteroid orders: a dobsonfly (order Megaloptera) and a giant lacewing and an owlfly (order Neuroptera). *Genome*, **2008**, 52(1), 31–38.

Hassan, M.A.; Shen, R.R.; Ali, M; Liu, X.Y. The first complete mitochondrial genomes of three dobsonfly species (Megaloptera: Corydalidae) from Pakistan with phylogenetic implications. *Revista Brasileira De Entomologia*. **2022**, 66(S1).

Hua, J.; Dong, P.; Xie, Q. The mitochondrial genome of *Protohermes concolorus* Yang et Yang 1988 (Insecta: Megaloptera: Corydalidae). *Mol. Biol. Rep*. **2009,** 36(7), 1757–1765.

Jiang, Y.L.; Yue, L.; Yang. F.; Gillung, J.P; Winterton, S.L.; Price, B.W.; Contreras-Ramos, A.; Hayashi, F.; Aspöck, U.; Aspöck, H.; Yeates, D.K.; Yang, D.; Liu, X.Y. Similar pattern, different paths: tracing the biogeographical history of Megaloptera (Insecta: Neuropterida) using mitochonadrial phylogenomics. *Cladistics*. **2022**, 38, 374–391.

Tu, Y.; Lin, A.; Jiang, Y; and Liu, X. Comparative mitochondrial genomics and phylogenetics among species of the Oriental dobsonfly genus *Neoneuromus* van der Weele, 1909 (Megaloptera: Corydalidae). *Journal of Asia-Pacific Entomology*. **2021**. 24, 1257–1265.

Xu, X.; Cheng, C.; Yan, B.; Su, E.; He, N. The complete mitochondrial genome of the *Neochauliodes fraternus* (Megaloptera: Corydalidae). *Mitochondrial DNA Part A* **2016**, 27(3), 2163–4.

Wang, Y.R.; Wang, Y.Y.; Chang, W.C.; Liu, X.Y.; Yang, D. Sequencing and analysis of the complete mitochondrial genome of the giant dobsonfly *Acanthacorydalis orientalis* (McLachlan) (Insecta: Megaloptera: Corydalidae). *Zool. Scr.* **2014**, *39*, 209–223
