# Supplementary figures and images for: Mitogenomic Insights into Temperature Adaptation: A Comparative Study of the Subfamily Corydalinae Davis, 1903 (Megaloptera: Corydalidae)
Source: Insects. 2025 Nov 10;16(11):1151. doi: 10.3390/insects16111151 (PMC12653108; doi:10.3390/insects16111151)

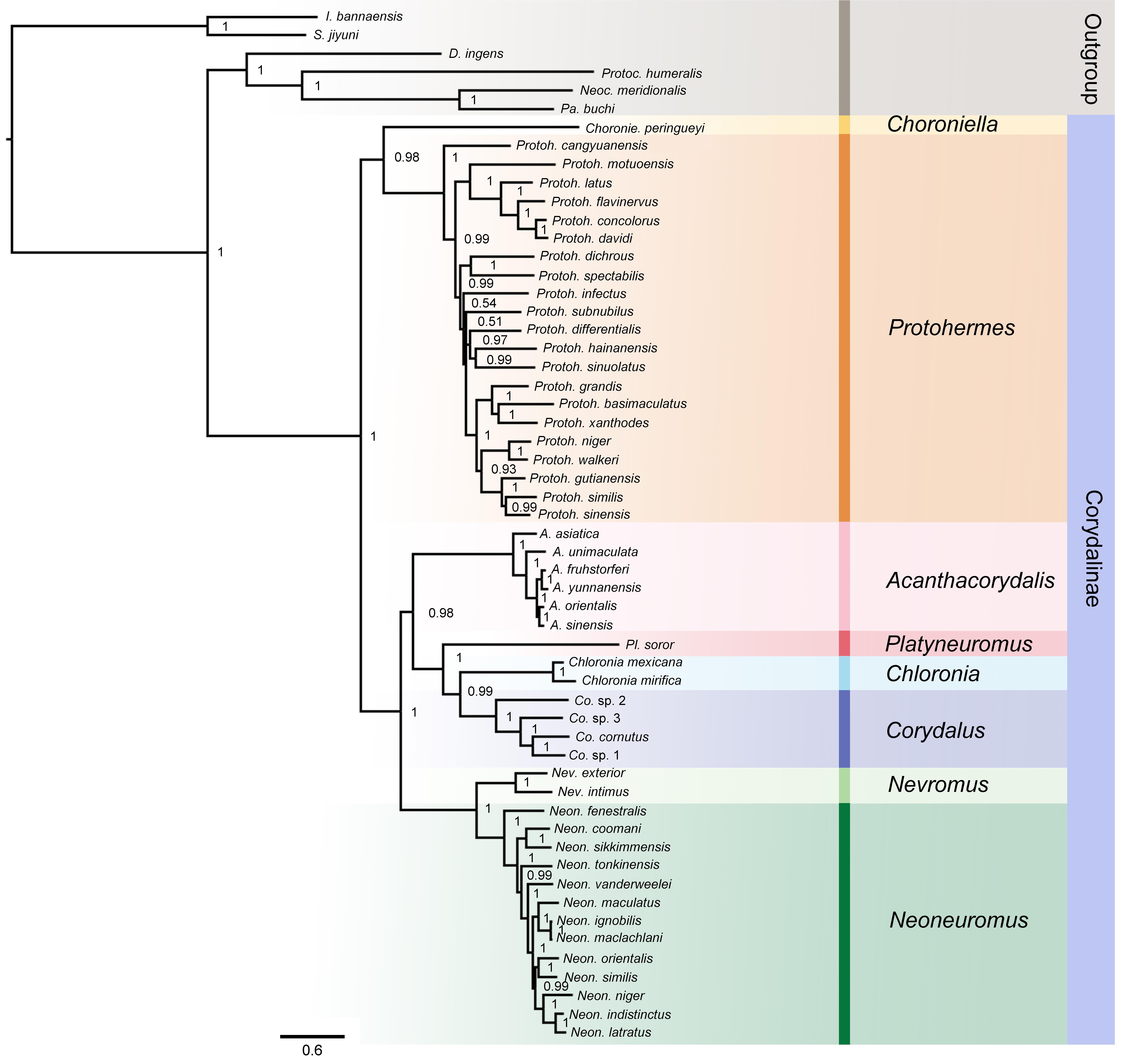

Supplement: Supplementary file 1 [file insects-16-01151-s001.zip › Figure S3.jpg]
